# A novel heterozygous mutation of CHD7 gene in a Chinese patient with Kallmann syndrome: a case report

**DOI:** 10.1186/s12902-021-00836-0

**Published:** 2021-09-25

**Authors:** Weiwei Xu, Weibin Zhou, Haiyang Lin, Dan Ye, Guoping Chen, Fengqin Dong, Jianguo Shen

**Affiliations:** 1 Department of Endocrinology and Metabolism, First Affiliated Hospital, School of Medicine, Zhejiang University, No.79, Qing-Chun Road, Zhejiang, 310003 Hangzhou China; 2 Department of Endocrinology, the Affiliated Wenling Hospital, Wenzhou Medical University, #333, S Chuan’an Road, Wenling, Zhejiang, 317500 China

**Keywords:** Kallmann syndrome, CHD7 gene, Idiopathic hypogonadotropic hypogonadism, Heterozygous mutation, Case report

## Abstract

**Background:**

Variants of chromodomain helicase DNA binding protein 7 (CHD7) gene are commonly associated with Kallmann syndrome (KS) and account for 5–6% of idiopathic hypogonadotropic hypogonadism (IHH) cases. Here we report a novel mutation of CHD7 gene in a patient with KS, which may contribute to the better understanding of KS.

**Case presentation:**

A 29-year-old male patient with KS and a chief complaint of delayed puberty for 13 years (Tanner B Stage< 4) was admitted to the Department of Endocrinology of the First Affiliated Hospital of Zhejiang University (Hangzhou, China) in September 2019. Dual-energy X-ray absorptiometry (DEXA) showed low bone density in both lumbar spine (L1 ~ L5 mean Z-score − 3.0) and femoral neck (Z-score − 2.7). Dynamic contrast-enhanced magnetic resonance imaging (MRI) of pituitary and contrast-enhanced computed tomography (CT) showed no abnormal findings. Ophthalmological evaluation showed that his both eyes showed exotropia, and no sight loss was noted. Heterozygous c.1619G > T mutation of TCD7 gene (p.G4856V) was detected, whereas none of his family members had this mutation. Human chorionic gonadotropin (HCG) and human menopausal gonadotropin (HMG) were injected for three times/week to treat idiopathic hypogonadotropic hypogonadism (IHH). After several months of therapy, the patient’s health condition improved. His testicles became larger, and his secondary sexual characteristics improved after treatment.

**Conclusion:**

Exploration of the novel splice-site mutation of CHD7 may further our current understanding of KS.

## Background

Kallmann syndrome (KS), characterized by hypogonadotropic hypogonadism and olfactory dysfunction, causes nearly 40–60% of idiopathic hypogonadotropic hypogonadism (IHH) in men [1]. KS is a rare genetic disorder in humans that results from defects in the production and/or action of hypothalamic peptide, controlling both male and female reproductive system [2]. The severity of gonadal dysgenesis and olfactory dysfunction of KS patients significantly vary among individuals, depending on different affected genes and modes of inheritance. Previous studies have demonstrated that KS is inherited by three classic inheritance patterns: X-linked recessive (i.e., caused by ANOS1 and DAX1 genes), autosomal dominant (i.e., caused by CHD7, FGFR1, and SOX10 genes), and autosomal recessive (i.e., caused by GNRH1 and KISS1 genes) [2, 3]. KS not only affects organs of the reproductive system, but also increases the risk of osteoporosis, cardiovascular disease, metabolic syndrome, psychological disorders, etc., remarkably threatening patient’s lifelong physical activity and mental health [4].

Chromodomain helicase DNA binding protein 7 (CHD7) gene is commonly associated with KS and IHH, and it accounts for 5–6% of IHH cases [5]. CHD7 protein is a transcriptional regulator that binds to enhancer elements in the nucleoplasm. CHD7 also functions as a positive regulator of ribosomal RNA (rRNA) biogenesis in the nucleolus [5]. CHD7 was originally identified as the causative gene in CHARGE syndrome [6]. CHARGE syndrome is a non-random clustering of congenital anomalies including coloboma of the eye, heart defects, choanal atresia, retarded growth and development, renital hypoplasia, dysmorphic ears, and/or hypoplasia or aplasia of the semicircular canals and deafness [7]. It was recently reported that CHARGE syndrome is caused by mutations in the CHD7 gene [8] . KS and CHARGE syndrome also share some similarities in clinical manifestations, such as olfactory disorder and hypogonadism, since the letter “G” in the CHARGE syndrome refers to gonadal dysgenesis, representing one of the minor diagnostic criteria of CHARGE syndrome [9].

In 2008, Kim et al. reported that CHD7 can cause KS without symptoms of CHARGE syndrome [10]. Since then, mutations of CHD7 gene, e.g., mutation in 1616 Tyr-Cys found by Costa-Barbosa in 2013, have been proven as the etiology of KS [11] . In contrast to the truncating CHD7 mutations that are observed typically in patients with CHARGE syndrome, patients with IHH harbor predominantly missense variants [12]. However, the underlying mechanism has remained elusive.

It is highly essential to perform genetic testing methods, such as Sanger sequencing, amplification refractory mutation system-polymerase chain reaction (ARMS-PCR), and differential display reverse transcription-PCR for precision medicine [2, 9], despite that they are difficultly popularized in clinical practice because of being costly and long duration of testing. The present research employed Sanger sequencing of the exons of genes related to genetic disorders. Globally, this is the first report that concentrated on the novel splice-site mutation of CHD7, aiming to make a significant progress for the pathogenic study of KS. Our results may supplement researches related to genetic disorders in China, improve the understanding of diseases, and promote the development of precision medicine, such as preimplantation genetic screening (PGS), preimplantation genetic diagnosis (PGD), prenatal screening, and prenatal diagnosis.

## Case presentation

### Patients’ clinical data

The patient found his penis smaller than usual 13 years ago, while he neglected it. In the recent 10 years, his penis and testicles never developed. He was previously treated with testosterone in a local hospital, while he found that treatment ineffective. Thus, he stopped testosterone therapy within 3 months before his admission to our hospital.

### Medical history

He was a full-term infant after a healthy pregnancy and delivery, with a birth weight of 3200 g. His gross and fine motor development were normal as usual in his childhood. He had a history of non-alcohol fatty liver disease, which was detected by ultrasound accidentally. His physical and mental health conditions were normal despite the existence of delayed puberty.

### Family history

His parents were healthy without consanguineous marriage. There were no similar diseases in his family, in addition to no history of special genetic diseases. He had a healthy younger sister, who had a biological child.

### Medical and imaging investigations

The patient’s height and weight were 174 cm and 72 kg, respectively. The diameter and length of his testicles and penis were 2 and 3.5 cm, respectively (Tanner B Stage< 4).

The values of biochemical parameters were as follows: total testosterone level of 24.16 ng/dL(142.39 ~ 923.14 ng/dL), luteinizing hormone (LH) level of 0.72 mIU/mL(0.57 ~ 12.07 mIU/mL), follicle-stimulating hormone (FSH) level of 1.05 mIU/mL(0.95 ~ 11.95 mIU/mL), sex hormone-binding globulin level of 14.7 nmol/L(17.1 ~ 77.6 nmol/L), and dehydroepiandrosterone sulfate level of 240.0 μg/dL(167.9 ~ 591.9 μg/dL).

Gonadotropin-releasing hormone (GnRH) stimulation test was revealed in Table 1.

**Table 1:** Result of GnRH (Gonadotropin-releasing hormone) stimulation test. LH(luteinizing hormone) and FSH(follicle-stimulating hormone) level 0,30,45,60,90,120 min after triptorelin acetate injected

	0	30 min	45 min	60 min	90 min	120 min
LH (mIU/mL)	1.16	1.89	1.92	2.20	2.24	2.44
FSH(mIU/mL)	0.72	2.41	2.54	2.76	3.04	3.13

Human chorionic gonadotropin (HCG) stimulation test showed that testosterone level was showed in Table 2.

**Table 2:** Result of HCG (human chorionic gonadotropin) stimulation test. Serum testosterone level 15 min before and 0,24,48,72 h after human chorionic gonadotropin injected

	-15 min	0 min	24 h	48 h	72 h
Testosterone(ng/dL)	17.58	19.48	44.35	54.98	57.0

Cortisol circadian rhythm and thyroid function showed no abnormalities.

Ultrasound displayed micro testis with a volume of less than 1 mL and multiple calcified lesions. The size of prostate was smaller than normal. Fatty liver was also detected by ultrasound.

Dynamic contrast-enhanced magnetic resonance imaging (MRI) of pituitary and contrast-enhanced computed tomography (CT) showed no abnormal findings.

Ophthalmological evaluation showed that his both eyes showed exotropia, and no sight loss was noted.

Ear, nose, and throat (ENT) evaluation showed that he had otitis media.

Dual-energy X-ray absorptiometry (DEXA) showed osteoporosis in both lumbar spine (L1 ~ L5 mean Z-score − 3.0) and femoral neck (Z-score − 2.7).

The bone age of the patient was about 16 years old interpreted with Greulich-Pyle methods, which showed a delay compared with his age.

### Genetic testing

**Fig. 1:**
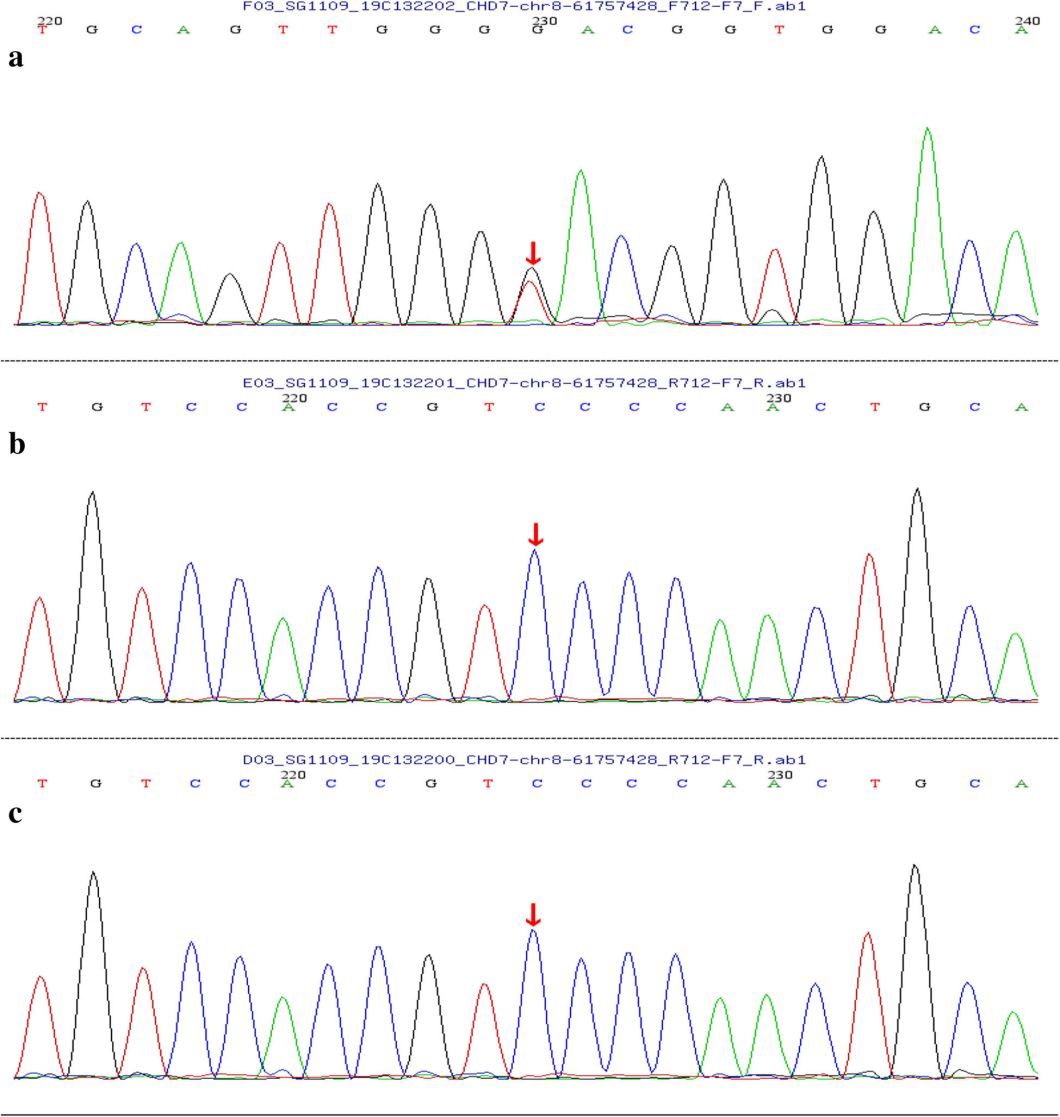
Sequence analysis of CHD7 gene. **a** heterozygous mutation of CHD7 gene can be detected, whereas **b** his father and **c** his mother don’t have this mutation

Primers were designed based on the sequence of sites validated by the CHD7 gene. Amplification was carried out with PCR, sequencing was performed with the ABI 3730XL sequencing device, and sequencing primers used the original PCR primers. Sanger sequencing was conducted using DNASTAR software, and mRNA alignment template was NM_003560 [13]. Gene testing was undertaken, in which a heterozygous mutation at c.1619G > T site of CHD7 gene (p.G4856V) was detected, which is a novel mutation site of CHD7 (Fig. 1).

### Treatment

Caltrate vitamin D and alendronate were prescribed to treat osteoporosis. The patient refused pulsatile GnRH therapy for economic concern. Human chorionic gonadotropin (HCG) and human menopausal gonadotropin (HMG) were injected for three times/week to treat IHH. After several months of therapy, the patient’s health condition improved. His testicles become larger and his secondary sexual characteristics improved after treatment.

## Discussion and conclusions

Previous studies have reported the embryologic development of KS. Olfactory neurons and GnRH neurons generate from the olfactory plate, and GnRH neurons migrate accompanied by olfactory bulb during process of embryogenesis. Thus, the manifestation of sexual dysgenesis and olfactory can co-exist due to a certain mutant gene. Several IHH-associated genes have been reported, such as ANOS1, SOX10, SEMA3A, etc. [10, 13, 14] .

In 2008, Kim et al. first reported 7 IHH patients with CHD7 mutations did not have major symptoms of CHARGE syndrome, which suggested CHD7 allelic variants were ascribed to KS and nIHH forms of IHH instead of CHARGE syndrome [10]. Bergman et al. also discovered 3 cases with CHD7 mutations out of 36 KS patients with respect to controversial results related to whether CHD7 mutations can cause KS or nIHH without features of CHARGE syndrome [13]. Besides, splice-site mutation c.1616 can cause KS [11]. We, for the first time, reported that mutation at 4856 can contribute to the development of KS, although the precise mechanism needs further research.

CHD7 protein is widely expressed in the olfactory epithelium, hypothalamus, and pituitary, suggesting that it may participate in the embryogenesis of the olfactory bulb and GnRH neurons [15]. Phenotypic analysis of families with members carrying deleterious CHD7 missense mutations showed a various phenotypes of CHD7-associated diseases; for instance, patients carrying the same mutation could have KS, nCHH or isolated anosmia [12, 16]. Additionally, phenotypes caused by CHD7 mutations include deafness and outer-ear abnormalities, hare lip/cleft palate, and cardiac abnormalities [12, 16, 17]. Consequently, it is necessary to diagnose these disorders in KS/CHH and improve genetic counseling. These observations indicate that a dose-function responsiveness of CHD7 mutations is related with the varied developmental processes and organ systems defects [17, 18]. It is observed that hypomorphic or dominant CHD7 alleles are higher probable to affect only the GnRH/HPG axis while null mutations affected multi-organ systems. The above-mentioned studies indicated that the ontogenesis of GnRH neurogenesis is particularly inclined to the mildest dysregulation of CHD7 function.

In addition to the existence of isolated KS in subjects harboring CHD7 mutations, it is confirmed that missense type mutations are more associated with partial loss of function, whereas truncating mutations (nonsense, frameshift and splice mutations) are more likely to be found in the traditional CHARGE syndrome [12, 16]. The precise molecular mechanisms underlying missense mutations causing IHH have been recently studied. Bouazoune et al. analyzed ATPase and nucleosome remodeling activity of mutant and WT CHD7 after he designed CHD7 missense mutations discovered in IHH [19]. They suggest that the milder missense CHD7 alleles associated with IHH may damage CHD7-related nucleosome remodeling function, which gives rise to the pathologic human phenotypes (e.g., IHH).

Typically, the majority of IHH patients respond to exogenous GnRH when a pulsatile regimen is administered, mimicking endogenous GnRH secretion with robust gonadotropin secretion [3]. In terms of therapy, boys with micropenis require testosterone or gonadotropin therapy during infancy to restore penile growth. Induction of fertility requires the use of either exogenous gonadotropin therapy or pulsatile GnRH therapy, which can be monitored by an experienced reproductive endocrinologist.

Molecular diagnosis is the most appropriate method to study the diagnosis of genetic disorders, such as KS and CHARGE syndrome. Next-generation sequencing (NGS) makes it possible to conduct high-throughput sequencing of multiple genes, along with all exons of genes related to genetic disorders. Thus, it can not only contribute to differential diagnosis for diseases resulted from known mutation sites, but also discover novel pathogenic mutations. Moreover, it is appropriate for differential diagnosis of genetic disorders with similar clinical features [19]. With development of new technologies, including PGD and PGT, particular pathogenesis-related (PR) protein genes can be deleted from family, therefore, genetic disorders are no longer incurable [6]. Further investigations should be conducted to explore more precise diagnosis and treatment approaches.

Our study is the first that reported a novel splice-site mutation of CHD7 gene. This novelty may contribute to the better understanding of KS, as well as developing further efficient therapies.

## Data Availability

Data sharing is not applicable to this article as no datasets were generated or analysed during the current study. The datasets used and/or analysed during the current study are available from the corresponding author on reasonable request.

## Abbreviations

CHD7: Chromodomain helicase DNA binding protein 7
KS: Kallmann syndrome
IHH: Idiopathic hypogonadotropic hypogonadism
ENT: Ear, nose, and throat
DEXA: Dual-energy X-ray absorptiometry
CT: Computed tomography
MRI: Magnetic resonance imaging
HCG: Human chorionic gonadotropin
HMG: Human menopausal gonadotropin
FSH: Follicle-stimulating hormone
LH: Luteinizing hormone
GnRH: Gonadotropin-releasing hormone

## References

[1] Bianco SD, Kaiser UB (2009). The genetic and molecular basis of idiopathic hypogonadotropic hypogonadism. Nat Rev Endocrinol.

[2] Maione L, Dwyer AA, Francou B, Guiochon-Mantel A, Binart N, Bouligand J, Young J (2018). GENETICS IN ENDOCRINOLOGY: Genetic counseling for congenital hypogonadotropic hypogonadism and Kallmann syndrome: new challenges in the era of oligogenism and next-generation sequencing. Eur J Endocrinol.

[3] Stamou MI, Georgopoulos NA (2018). Kallmann syndrome: phenotype and genotype of hypogonadotropic hypogonadism. Metabolism.

[4] Garrido Oyarzún MF, Castelo-Branco C (2016). Sexuality and quality of life in congenital hypogonadisms. Gynecol Endocrinol.

[5] Balasubramanian R, Crowley WF (2017). Reproductive endocrine phenotypes relating to CHD7 mutations in humans. Am J Med Genet C Semin Med Genet.

[6] Young J, Xu C, Papadakis GE, Acierno JS, Maione L, Hietamäki J, Raivio T, Pitteloud N (2019). Clinical Management of Congenital Hypogonadotropic Hypogonadism. Endocr Rev.

[7] van Ravenswaaij-Arts C, Martin DM (2017). New insights and advances in CHARGE syndrome: Diagnosis, etiologies, treatments, and research discoveries. Am J Med Genet C Semin Med Genet.

[8] Hefner MA, Fassi E (2017). Genetic counseling in CHARGE syndrome: Diagnostic evaluation through follow up. Am J Med Genet C Semin Med Genet.

[9] Topaloğlu AK (2017). Update on the Genetics of Idiopathic Hypogonadotropic Hypogonadism. J Clin Res Pediatr Endocrinol.

[10] Kim HG, Kurth I, Lan F, Meliciani I, Wenzel W, Eom SH, Kang GB, Rosenberger G, Tekin M, Ozata M (2008). Mutations in CHD7, encoding a chromatin-remodeling protein, cause idiopathic hypogonadotropic hypogonadism and Kallmann syndrome. Am J Hum Genet.

[11] Costa-Barbosa FA, Balasubramanian R, Keefe KW, Shaw ND, Al-Tassan N, Plummer L, Dwyer AA, Buck CL, Choi JH, Seminara SB (2013). Prioritizing genetic testing in patients with Kallmann syndrome using clinical phenotypes. J Clin Endocrinol Metab.

[12] Marcos S, Sarfati J, Leroy C, Fouveaut C, Parent P, Metz C, Wolczynski S, Gérard M, Bieth E, Kurtz F (2014). The prevalence of CHD7 missense versus truncating mutations is higher in patients with Kallmann syndrome than in typical CHARGE patients. J Clin Endocrinol Metab.

[13] Bergman JE, de Ronde W, Jongmans MC, Wolffenbuttel BH, Drop SL, Hermus A, Bocca G, Hoefsloot LH, van Ravenswaaij-Arts CM (2012). The results of CHD7 analysis in clinically well-characterized patients with Kallmann syndrome. J Clin Endocrinol Metab.

[14] Balasubramanian R, Choi JH, Francescatto L, Willer J, Horton ER, Asimacopoulos EP, Stankovic KM, Plummer L, Buck CL, Quinton R (2014). Functionally compromised CHD7 alleles in patients with isolated GnRH deficiency. Proc Natl Acad Sci U S A.

[15] Bartels CF, Scacheri C, White L, Scacheri PC, Bale S (2010). Mutations in the CHD7 gene: the experience of a commercial laboratory. Genet Test Mol Biomarkers.

[16] Xu C, Cassatella D, van der Sloot AM, Quinton R, Hauschild M, De Geyter C, Flück C, Feller K, Bartholdi D, Nemeth A (2018). Evaluating CHARGE syndrome in congenital hypogonadotropic hypogonadism patients harboring CHD7 variants. Genet Med.

[17] Bergman JE, Janssen N, Hoefsloot LH, Jongmans MC, Hofstra RM, van Ravenswaaij-Arts CM (2011). CHD7 mutations and CHARGE syndrome: the clinical implications of an expanding phenotype. J Med Genet.

[18] Bouazoune K, Kingston RE (2012). Chromatin remodeling by the CHD7 protein is impaired by mutations that cause human developmental disorders. Proc Natl Acad Sci U S A.

[19] Cangiano B, Swee DS, Quinton R, Bonomi M. Genetics of congenital hypogonadotropic hypogonadism: peculiarities and phenotype of an oligogenic disease. Hum Genet. 2020. 10.1007/s00439-020-02147-1. 10.1007/s00439-020-02147-1 32200437

